# Psammaceratin A: A Cytotoxic Psammaplysin Dimer Featuring an Unprecedented (2Z,3Z)-2,3-Bis(aminomethylene)succinamide Backbone from the Red Sea Sponge *Pseudoceratina arabica*

**DOI:** 10.3390/md19080433

**Published:** 2021-07-29

**Authors:** Diaa T. A. Youssef, Hani Z. Asfour, Lamiaa A. Shaala

**Affiliations:** 1 Department of Natural Products, Faculty of Pharmacy, King Abdulaziz University, Jeddah 21589, Saudi Arabia; 2 Department of Medical Parasitology, Faculty of Medicine, Princess Al-Jawhara Center of Excellence in Research of Hereditary Disorders, King Abdulaziz University, Jeddah 21589, Saudi Arabia; hasfour@kau.edu.sa; 3 Natural Products Unit, King Fahd Medical Research Center, King Abdulaziz University, Jeddah 21589, Saudi Arabia; 4 Department of Medical Laboratory Sciences, Faculty of Applied Medical Sciences, King Abdulaziz University, Jeddah 21589, Saudi Arabia; 5 Suez Canal University Hospital, Suez Canal University, Ismailia 41522, Egypt

**Keywords:** Red Sea sponge, *Pseudoceratina arabica*, marine alkaloids, psammaplysin dimer, psammaceratin A, psammaplysin A, cell lines’ growth inhibition

## Abstract

Bioassay-guided partition of the extract of the Red Sea sponge *Pseudoceratina arabica* and HPLC purification of the active fraction gave a psammaplysin dimer, psammaceratin A (**1**), along with psammaplysin A (**2**). The dimer comprises two units of psammaplysin A (**2**) connected via the terminal amines with an unprecedented (2*Z*,3*Z*)-2,3-bis(aminomethylene)succinamide moiety, and it represents the first dimer to be identified among the psammaplysin family. Data from 1D- and 2D-NMR and HRMS supported the chemical structures of the compounds. Psammaceratin A (**1**) and psammaplysin A (**2**) exhibited significant growth inhibition of HCT 116, HeLa, and MBA-MB-231 cells down to 3.1 μM.

## 1. Introduction

Verongid sponges are considered a main source of bioactive brominated alkaloids that originate from bromotyrosine [1,2,3]. These marine alkaloids include a large and structurally diverse class of compounds such as psammaplysins (spirooxepinisoxazolines), bastadins, spirocyclohexadienylisoxazolines, oximes, and others [1,2,3]. Compounds with the dibrominated spirooxepinisoxazoline moiety are reported under different names, including psammaplysins [2,3,4,5,6,7,8,9,10,11,12,13], ceratinamides [12,13,14], ceratinadins [15], and frondoplysins [16]. Spirooxepinisoxazoline derivatives originate mainly from members of the order Verongiida [4,5,6,7,8,9,10,11,12,13], with only two sponges belonging to the Dictyoceratida order [14,15,16]. Currently, 43 compounds with a spirooxepinisoxazoline skeleton have been reported, including psammaplysins A–Z, 19-hydroxy derivatives of several psammaplysins, psammaplysin K-dimethyl acetal [2,3,4,5,6,7,8,9,10,11,12,13], ceratinadins E and F [15], ceratinamide A, 19-hydroxyceratinamde A and ceratinamide B [11,12], and frondoplysins A and B [16]. Out of the 43 compounds with a spirooxepinisoxazoline moiety, 40 compounds are documented from the order Verongiida (members of the genera *Aplysinella, Psammaplysinella*, *Pseudoceratina*, and *Suberea*) [4,5,6,7,8,9,10,11,12,13,15]. The remaining three candidates are reported from *Hyattella* sp. [14]. and *Dysidea frondosa* [16] of the order Dictyoceratida.

Psammaplysin A was the first reported compound with the spirooxepinisoxazoline backbone [4], and it includes, in addition to the substituted spirooxepinisoxazoline backbone, another dibrominated bromotyrosine-derived unit named moloka’iamine [2], connected together via an amidic linkage [13]. Psammaplysins’ derivatives have been associated with diverse pharmacological properties, such as cytotoxic [5,11,13], antimigratory [17], antimalaria [8,10,14,15], antiviral [6], antifouling [14], antimicrobial [18], antioxidant [16], and immunosuppressive [6] activities. 

In a continuation of our survey of Verongid sponges in the Red Sea for bioactive chemical entities [13,19,20,21,22], bioassay-guided fractionation of the cytotoxic fraction of the methanolic extract of *P. arabica* afforded a novel symmetrical psammaplysin dimer, psammaceratin A (**1**), and the known psammaplysin A (**2**) [4]. Psammaceratin A (**1**) possesses an unprecedented moiety, (2*Z*,3*Z*)-2,3-bis(aminomethylene)succinamide, linking symmetrically through the terminal amino groups of two units of psammaplysin A with C-21/C-21′ of the substituted 2,3-bis(aminomethylene)succinamide part of the molecule.

## 2. Results and Discussion

### 2.1. Structure of Psammaceratin A (***1***)

**Figure 1:**
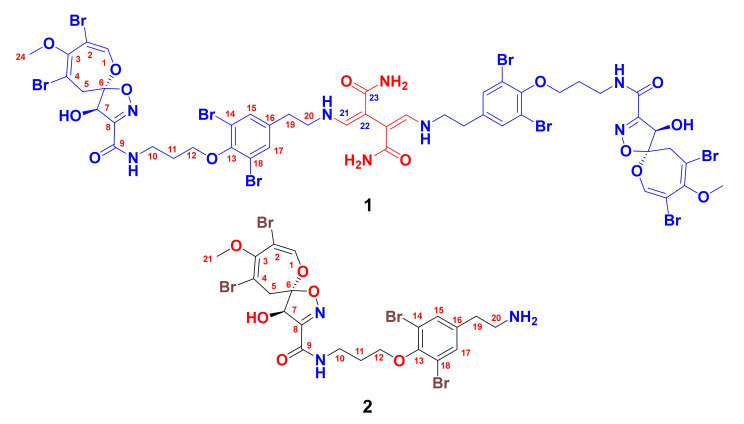
Chemical structures of **1** and **2**.

**Figure 2:**
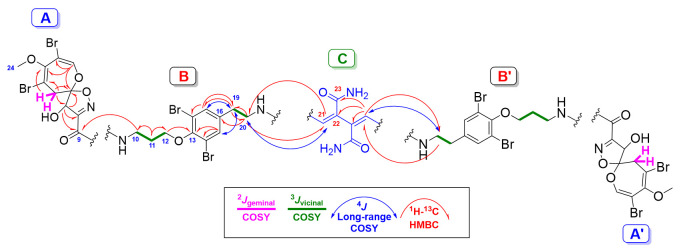
Structural subunits and significant COSY and HMBC of **1**.

Psammaceratin A (**1**) (Figure 1) was obtained as an optically active ([α]$\begin{array}{c} 25\\ D \end{array}$ = −59°) compound. The existence of the ion peaks at *m/z* 1616.6, 1618.6, 1620.6, 1622.6, 1624.6, 1626.6, 1628.6, 1630.6, and 1632.6 (1:8:28:56:70:56:28:8:1, [M + Na]^+^) in the positive ESIMS of **1** supported the presence of eight bromine atoms. The molecular formula of **1** was established as C_48_H_50_Br_8_N_8_O_14_ by positive HRESIMS (*m/z* 1624.6732, C_48_H_50_^79^Br_4_^81^Br_4_N_8_O_14_Na, [M + Na]^+^) (Figure S1), suggesting 24 degrees of unsaturation. Its ^13^C NMR spectrum exhibited 22 resonances, corresponding to 24 carbons, signifying the presence of a symmetrical dimer (Table 1). Investigation of the complete NMR spectra of **1** including ^1^H (Figures S2 and S3), ^13^C (Figure S4), DEPT (Figure S5), ^1^H-^1^H COSY (Figure S6), and multiplicity-edited HSQC (Figure S7) experiments confirmed the existence of five structural subunits in **1**, including two similar pairs (A and A′, B and B′) together with an unprecedented moiety (fragment C) (Table 1 and Figure 2).

The first similar subunits (A and A′) in **1** are assigned as 2,3,4,7,9-penta-substituted spirooxepinisoxazoline moieties. These assignments are supported by the ^1^H and ^13^C NMR resonances at δ_H/C_ 7.13 (s)/146.8 (CH, C-1/1′), 104.4 (qC, C-2/2′), 1549.9 (qC, C-3/3′), 104.6 (qC, C-4/4′), 3.38 (d, ^2^*J* = 16.2 Hz) and 3.05 (d, ^2^*J* = 16.2 Hz)/38.3 (CH_2_, C-5/5′), 120.9 (qC, C-6/6′), 4.98 (s)/80.5 (CH, C-7/7′), 158.8 (qC, C-8/8′), 160.7 (qC, C-9/9′), and 3.64 (s)/59.4 (CH_3_, C-25/25′). These signals are characteristic of the presence of two similar moieties, namely, 2,3,4,7,9-penta-substituted spirooxepinisoxazoline [2,3,4,5,6,7,8,9,10,11,12,13]. The locations of the bromine atoms, OCH_3_, and OH moieties on the spirooxepinisoxazoline moieties at C-2/2′, C-4/4′, C-3/3′, and C-7/7′, respectively, were supported by HMBC cross-peaks from H-1/1′ to C-2/2′, C-3/3′, and C-6/6′; from H_2_-5/5′ to C-3/3′, C-4/4′, and C-6/6′; from H-7/7′ to C-6/6′, C-8/8′, and C-9/9′; and from H_3_-24/24′ to C-3/3′ (Table 1 and Figure 2). Furthermore, these HMBC correlations supported the assignment of the quaternary carbons in subunits A and A′ and complete the unequivocal assignment of the first subunits (A and A′) of **1** (Figure 2).

The second identical subunits of **1** (B and B′) (Figure 2) are assigned as two *N,N*-disubstituted moloka’iamine moieties. Two spin–spin coupling systems from H_2_-10/10′ to H_2_-12/12′ and between H_2_-19/19′ and H_2_-20/20′ were traced from the COSY experiment. The δ_H/C_ 3.60 (t, *J* = 6.5 Hz)/38.1 (CH_2_, C-10/10′), 2.12 (quin., *J* = 6.5 Hz)/30.6 (CH_2_, C-11/11′), 4.05 (t, *J* = 6.5 Hz)/72.2 (CH_2_, C-12/12′), 153.4 (qC, C-13/13′), 119.4 (2 × qC, C-14/14′ and C-18/18′), 7.43 (s)/134.8 (2 × CH, C-15/15′ and C-17/17′), 138.1 (qC, C-16/16′), 2.90 (t, *J* = 7.2 Hz)/36.1 (CH_2_, C-19/19′), and 3.69 (t, *J* = 7.2 Hz)/52.6 (CH_2_, C-20/20′) suggested the existence of two moloka’iamine moieties, substituted on their terminal amines [14,15]. There was a significant downfield shift of the ^13^C NMR signals of the methylenic moieties C-19/19′ and C-20/20′ and the ^1^H NMR signals of H_2_-19/19′ in **1** in comparison with psammaplysin A (**2**), which possesses a free terminal amine at C-20. The change in the NMR shifts of these signals is attributed to the substitution of the free amines at C-20/20′ in **1** by a substituted succinimide moiety as discussed below (Table 2). The HMBC correlations (Table 1 and Figure 2 and Figure S8) from H_2_-10/10′ to C-11/11′ and C-12/12′; from H_2_-11/11′ to C-10/10′ and C-12/12′; from H_2_-12/12′ to C-10/10′, C-11/11′, and C-13/13′ (δ_C_ 153.4); from H-15/15′ and H-17/17′ to C-13/13′, C-14/14′, C-18/18′ (δ_C_ 119.4), and C-19/19′ (δ_C_ 36.1); from H_2_-19/19′ to C-16/16′ (δ_C_ 138.1), C-17/17′, and C-20/20′; and, finally, from H_2_-20/20′ to C-16/16′ and C-19/19′ secured the structure of the subunits B and B′ of **1**. 

The connections between the subunits A and B and between A′ and B′ through the amidic linkages C-9−*N* and C-9′−*N*′ are reinforced by HMBC correlations from H_2_-10/10′ (δ_H_ 3.60) to C-9/9′ (δ_C_ 160.7) and from H-7/7′ (δ_H_ 4.98) to C-9/9′ (δ_C_ 160.7) (Table 1 and Figure 2 and Figure S8).

The sum of the elements of the assigned subunits A, A′, B, and B′ was counted for C_42_H_44_Br_8_N_6_O_12_ and for 20 degrees of unsaturation. The remaining elements of C_6_H_6_N_2_O_2_ (Fragment C) were counted for the remaining four degrees of unsaturation in **1**. These elements C_6_H_6_N_2_O_2_ are assigned as 2,3-bis(aminomethylene)succinamide (Figure 2) based on the remaining NMR resonances at δ_H/C_ (CH, 7.54/157.1, C-21/21′), 119.2 (qC, C-22/22′), and 171.1 (qC, C-23/23′) (Table 1 and Figure 2 and Figure S8). This assignment was established by HMBC from H_2_-20/20′ to C-21/21′, from H-21/21′ to C-20/20′, from H-21/21′ to C-22/22′, and from H-21/21′ to C-23/23′, completing the assignment of fragment C. 

The linkage of subunit C with structural parts B and B′ through the terminal amines and C-21/21′ (NH-C-21 and NH-C-21′) is supported by the long-range COSY couplings (^4^*J*) between H_2_-20/20′ (δ_H_ 3.69) and H-21/21′ (δ_H_ 7.54), as well by HMBC correlations from H_2_-20/20′ (δ_H_ 3.69) to C-21/21′ (δ_C_ 157.1) and from H-21/21′ (δ_H_ 7.54) to C-20/20′ (δ_C_ 52.6) (Table 1 and Figure 2 and Figure S8), completing the degrees of unsaturation and the planar structure of psammaceratin A. 

The substitution of the terminal amines in **1** with 2,3-bis(aminomethylene)succinamide moiety caused a significant and expected ^13^C NMR downfield shift of the carbons of the ethylene moieties (C-19/19′ and C-20/20′) from δ_C_ 31.8 (C-19) and δ_C_ 40.0 (C-20) in psammaplysin A (**2**) to δ_C_ 36.1 (C-19/19′) and δ_C_ 52.6 (C-20/20′) (Δδ_C_ = +5.3 and +12.6 ppm, respectively) in **1** (Table 2). An additional significant and expected downfield shift of H_2_-20 from 3.18 ppm (in **2**) to 3.69 ppm in **1** (Δδ_H_ = +0.51 ppm) was observed, confirming the effect of the substitution of the terminal amines in psammaplysin derivatives [11] (Table 2).

**Figure 3:**
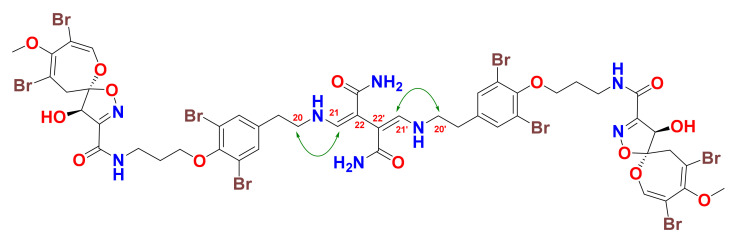
Significant NOESY correlations at the Z-configured Δ^21,22^ and Δ^21^′^,22^′ in **1**.

**Figure 4:**
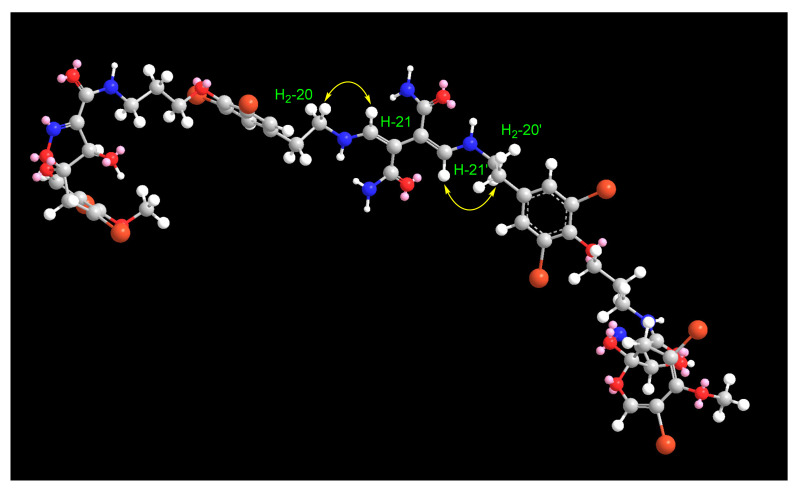
An MM2 energy-minimized model of **1** displaying NOEs between H_2_-20/20′ and H-21/21.

The Δ^21,22^ and Δ^21^^′,22^^′^ configurations of the 2,3-bis(aminomethylene)succinamide moiety were assigned as *Z* and *Z* based on the presence of the NOE correlations between H_2_-20/20′ and H-21/21′ in the NOESY experiment (Figure 3 and Figure S9). In an MM2 energy-minimized drawing of **1** (Figure 4), strong NOE correlations are expected between H_2_-20 and H-21 and between H-20′ and H-21′ (Figure 3 and Figure 4). Accordingly, the *Z* configurations at Δ^21,22^ and Δ^21^^′,22^^′^ are confirmed. 

Compound **1** displayed optical activity with a similar sign and magnitude ([α]$\begin{array}{c} 25\\ D \end{array}$ = −59°) to that of reported psammaplysins [2,4,6,8,9,11,13]. Therefore, it is more likely that psammaceratin A possesses the same biosynthetic path and shares similar stereochemistry at C-6/6′ and C-7/7′ with reported psammaplysins [2,4,6,8,9,11,13]. In addition, the sign and magnitude of the optical rotation of psammaceratin A are closely correlated to reported values [2,4,6,8,9,11,13]. The absolute configurations at C-6 and C-7 in psammaplysin A (**2**) were recently verified as 6*R* and 7*R* [23]. Therefore, we anticipate that psammaceratin A (**1**) shares the same absolute configurations of 6*R*,7*R* and 6′*R*,7′*R* with the parent compound, psammaplysin A [23]. Thus, psammaceratin A was assigned as (2*Z*,3*Z*)-2,3-bis(((3,5-dibromo-4-(3-((4*R*,5*R*)-8,10-dibromo-4-hydroxy-9-methoxy-1,6-dioxa-2-azaspiro[4.6]undeca-2,7,9-triene-3-carboxamido)propoxy)phenethyl)amino)methylene)succinamide.

In an MTT assay [13,24], psammaceratin A (**1**) displayed the highest activity against HCT 116 cells with an IC_50_ value of 3.1 μM. On the contrary, psammaplysin A (**2**), with its free terminal amine moiety, was less active towards HCT 116, with an IC_50_ value of 5.1 μM. On the other hand, compound **2** was more active against MDA-MB-231 (IC_50_ = 3.90 μM), while **1** was less active against this cell line (IC_50_ = 5.25 μM). These data suggest that MDA-MB-231 and HCT 116 have high sensitivities towards **1** and **2**, respectively. Finally, psammaceratin A (**1**) and psammpalysin A (**2**) displayed close and similar activity towards HeLa cells (IC_50_ = 8.50–9.40 μM) (Table 3) suggesting lower sensitivity of this cell line towards **1** and **2**. Thus, psammaceratin A and psammaplysin A are considered as potential leads for the establishment of novel anticancer entities.

### 2.2. Structure of Psammaplysin A (***2***)

Psammaplysin A (**2**) (Figure 1) was identified by interpretation of its NMR and MS data and by comparison of its NMR data with the literature [4]. 

## 3. Materials and Methods

### 3.1. General Experimental Procedures 

Optical rotations were acquired on a digital DIP-370 polarimeter (JASCO). The UV spectra were measured on a Hitachi 300 spectrometer. NMR data were acquired on a Bruker Avance DRX 600 MHz spectrometer using CD_3_OD as the solvent. Positive ion HRESIMS spectra were collected on a Thermo LTQ Orbitrap XL mass spectrometer. A SiO_2_ RP HPLC column (Merck, 70–230 mesh ASTM) and Sephadex LH 20 (0.25–0.1 mm, Pharmacia) were used for chromatography. An HPLC column (AR II Cosmosil, Waters, 250 × 10 mm, 5 μm) was used for purification of the compounds. 

### 3.2. Biological Materials

The Red Sea *Pseudoceratina arabica* (Keller, 1883) was harvested by hand via scuba down to −17 m from Anas Reef, Obhur at the Saudi Red Sea coast (N 021°39′17.5′′, E 038°52′26.3′′). The sponge consists of an encrusting mass of 1–2 cm with a conulose surface of yellowish green color underwater and greenish yellow interior. The sponge starts to turn blackish green in color after exposure to air. After storage in 70% ethanol solution, it turns completely into a black mass. The conules on the surface of the sponge are bluntly rounded, about 2–5 mm apart, and are of rubbery and compressible consistency. The specimen fragment measured about 12.0 × 5.0 × 1.5 cm. The sponge’s skeleton contains spare unequal fibers containing only pith. The outline branching is irregular, and the thickness measures 80–300 μm. The specimen corresponded to an Eritrean Red Sea specimen of *P. arabica*. The voucher was stored in the Zoological Museum’s collection at Amsterdam University under the code RMNHPOR 9161. Another specimen was stored at King Abdulaziz University under code #KSA-58. 

### 3.3. Purification of ***1*** and ***2***

Freeze-dried sponge materials (230 g) were extracted thrice with 1000 mL MeOH. The combined extracts were dried under vacuum to afford 5.29 g of viscous residue. The extract was dissolved in MeOH–H_2_O (6:4) and successively extracted with hexane, CH_2_Cl_2_, and EtOAc. The cytotoxic CH_2_Cl_2_ extract (2.1 g) was chromatographed over a SiO_2_ VLC column using hexane/EtOAc/MeOH gradients to afford five fractions (Fr-1–Fr-5). Fractionation of the cytotoxic fraction (Fr-3) (270 mg) on a Sephadex LH 20 column with MeOH gave four major subfractions (Fr-3A–Fr-3C). The cytotoxic fraction Fr-3B (45 mg) was purified on an ODS HPLC column using 80% MeOH to yield compounds **1** (5.3 mg) and **2** (2.7 mg).

### 3.4. Spectral Data of the Compounds

Psammaceratin A (**1**): pale yellow solid; [α]$\begin{array}{c} 25\\ D \end{array}$ = −59° (c 0.1, MeOH); NMR data: Table 1; HRESIMS m/z 1624.6732, (calcd for C_48_H_50_^79^Br_4_^81^Br_4_N_8_O_14_Na, [M + Na]^+^, 1624.6729). 

Psammaplysin A (**2**) was identified by analyses of its 1D and 2D NMR data and by comparison of its spectroscopic data to those in the literature [4]. 

### 3.5. Cytotoxicity of the Compounds

#### 3.5.1. Preparation of Cell Lines and Cell Culture

The human cell lines HCT116 (colorectal carcinoma, ATCC CCL-247), MDA-MB-231 (triple-negative breast cancer, *ATCC* HTB-26), and HeLa (human cervical carcinoma, *ATCC* CCL-2) were used in this investigation. For MDA-MB-231, DMEM with 1% penicillin–streptomycin and 10% FBS was used, while RPMI 1640 medium with 10% FBS and 1% penicillin–streptomycin was used for culturing HCT116 and HeLa cells. All cells were incubated at 37 °C with 5% CO_2_ and 95% humidity.

#### 3.5.2. Cytotoxicity and Antiproliferative Activity

The evaluation of the cytotoxicity of the compounds was carried out via MTT assay as previously described [13,24]. Briefly, cells were incubated at 37 °C overnight in 5% CO_2_/air, followed by the addition of the compounds at the top row of a 96-well microtiter plate and descending serial dilution (1:4) of the concentration. The cells were incubated for 72 h with the compounds. Subsequently, the cell viability was estimated at 490 nm on a Molecular Devices Emax microplate reader using the Cell Titer 96 AQueous non-radioactive cell proliferation protocol. The IC_50_ values of the compounds (expressed in micromoles) were evaluated using the program SOFTmax PRO. 5-Fluorouracil (5-FU) and DMSO were used as positive negative controls.

## 4. Conclusions

Bioassay-directed fractionation of the cytotoxic extract of the Red Sea sponge *Pseudoceratina arabica* afforded an unprecedented psammaplysin dimer, psammaceratin A (**1**), along with psammaplysin A (**2**). Psammaceratin A, with its unique (2*Z*,3*Z*)-2,3-bis(aminomethylene)succinamide backbone connecting two units of psammaplysin A, represents the first dimer of this type within the psammaplysin family. This previously unknown functional group, 2,3-bis(aminomethylene)succinamide, offers a novel synthetic moiety that could be utilized as an isostere in synthetic chemistry, as a novel connecting moiety, or for other design and derivatization purposes. Accordingly, psammaceratin A and psammaplysin A are potential scaffolds for the development of novel antitumor leads. 

## Figures and Tables

**Table 1:** NMR data of **1** (600 MHz for ^1^H and 150 for ^13^C, CD_3_OD).

No.	δ_C_	δ_H_	HMBC	NOESY
1, 1′	146.8, CH	7.13 (s)	C-2/2′, C-3/3′, C-6/6′	H_2_-11/11′
2, 2′	104.4, qC			
3, 3′	149.9, qC			
4, 4′	104.6, qC			
5a,5a’	38.3, CH_2_	3.38 (d, 16.2)	C-3/3′, C-4/4′, C-6/6′, C-7/7′	H-5b/5b′, H-7/7′
5b,5b’		3.05 (d, 16.2)	C-3/3′, C-4/4′, C-6/6′	H-5a/5a′, H-7/7’
6, 6′	120.9, qC			
7, 7′	80.5, CH	4.98 (s)	C-6/6′, C-8/8′, C-9/9′	H-5a/5a′, H-5b/5b′
8, 8′	158.8, qC			
9, 9′	160.7, qC			
10, 10′	38.1, CH_2_	3.60 (t, 6.5)		H_2_-11, H_2_-12
11, 11′	30.6, CH_2_	2.12 (quin., 6.5)		H-5b/5b′, H_2_-9/9′, H_2_-10/10′, H_2_-12/12′
12,12′	72.2, CH_2_	4.05 (t, 6.5)		H_2_-10/10′, H_2_-11/11′
13, 13′	153.4, qC			
14, 14′	119.4, qC			
15, 15′	134.8, CH	7.43 (s)	C-13/13′, C-14/14′, C-16/16′, C-18/18′, C-19/19′	H_2_-19/19′
16, 16′	138.1, qC			
17, 17′	134.8, CH	7.43 (s)	C-13/13′, C-14/14′, C-16/16′, C-18/18′, C-19/19′	H_2_-19/19′
18, 18′	119.4, qC			
19, 19′	36.1, CH_2_	2.90 (t, 7.2)	C-15/15′, C-16/16′, C-17/17′, C-20/20′	H_2_-20/20′
20, 20′	52.6, CH_2_	3.69 (t, 7.2)	C-16/16′, C-19/19′, C-21/21′	H_2_-19/19′
21, 21′	157.1, CH	7.54 (s)	C-20/20′, C-22/22′, C-23/23′	
22, 22’	119.2, qC			
23, 23′	171.1, qC			
24, 24′	59.4, CH_3_	3.64 (s)	C-3/3′	

**Table 2:** Comparison of partial NMR data of **1** and **2** (CD_3_OD).

Position	Psammaplysin A (2) *		Psammaceratin A (1)		Δδ (ppm)	
	δ_C_ *	δ_H_ *	δ_C_	δ_H_	Δδ_H_	Δδ_C_
19/19′	31.8	2.93	36.1	2.90	−0.03	+5.3
20/20′	40.0	3.18	52.6	3.69	+0.51	+12.6

(*) Data from this study.

**Table 3:** Cytotoxic activities of **1** and **2**.

Compound		IC_50_ (μM)	
	MDA-MB-231	HeLa	HCT 116
**1**	3.90 ± 0.20	8.50 ± 0.81	5.10 ± 0.41
**2**	5.25 ± 0.48	9.40 ± 0.89	3.10 ± 0.28
5-FU	13.0 ± 0.30	12.3 ± 0.25	4.60 ± 0.23

## Data Availability

Data is contained within the article or supplementary material at www.mdpi.com/article/10.3390/md19080433/s1.

## Supplementary Materials

The following are available online at https://www.mdpi.com/article/10.3390/md19080433/s1, Figures S1–S9: HRESIMS, ^1^HNMR, ^13^C NMR, DEPT, COSY, HSQC, and HMBC, and NOESY spectra of psamaceratin A (**1**).
