# Supplementary material for: Psammaceratin A: A Cytotoxic Psammaplysin Dimer Featuring an Unprecedented (2Z,3Z)-2,3-Bis(aminomethylene)succinamide Backbone from the Red Sea Sponge Pseudoceratina arabica
Source: Mar Drugs. 2021 Jul 29;19(8):433. doi: 10.3390/md19080433 (PMC8399316; doi:10.3390/md19080433)

Supporting Information

Figure S1. HRESIMS spectrum of psammaceratin A (**1**) ··········· 1
Figure S2. 600 MHz ^{1}H NMR spectrum of psammaceratin A (**1**) ··········· 2
Figure S3. Expansion of ^{1}H NMR spectrum of psammaceratin A (**1**) ··········· 3
Figure S4. 150 MHz ^{13}C NMR spectrum of psammaceratin A (**1**) ··········· 4
Figure S5. DEPT spectrum of psammaceratin A (**1**) ··········· 5
Figure S6. ^{1}H-^{1}H COSY spectrum of psammaceratin A (**1**) ··········· 6
Figure S7. Multiplicity-edited HSQC spectrum of psammaceratin A (**1**) ··········· 7
Figure S8. ^{1}H-^{13}C HMBC spectrum of psammaceratin A (**1**) ··········· 8
Figure S9. NOESY spectrum of psammaceratin A (**1**) ··········· 9

Figure S1. HRESIMS spectrum of psammaceratin A (**1**).

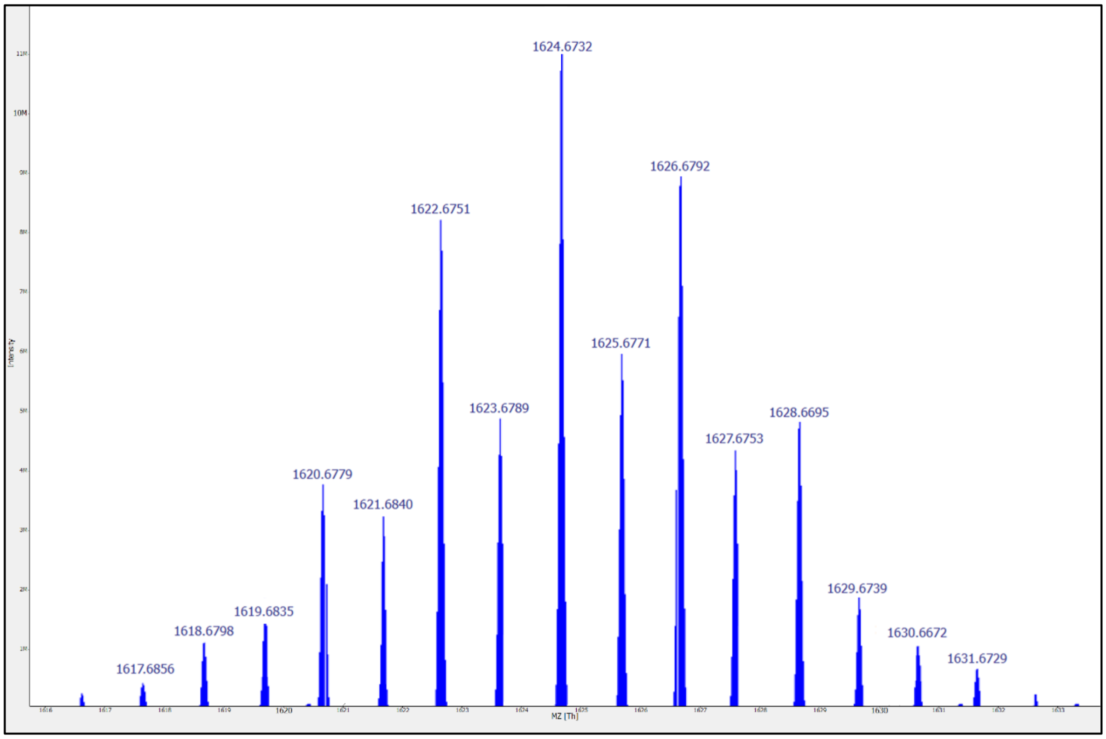

Figure S2. 600 MHz ^{1}H NMR spectrum of psammaceratin A (**1**) (MeOD).

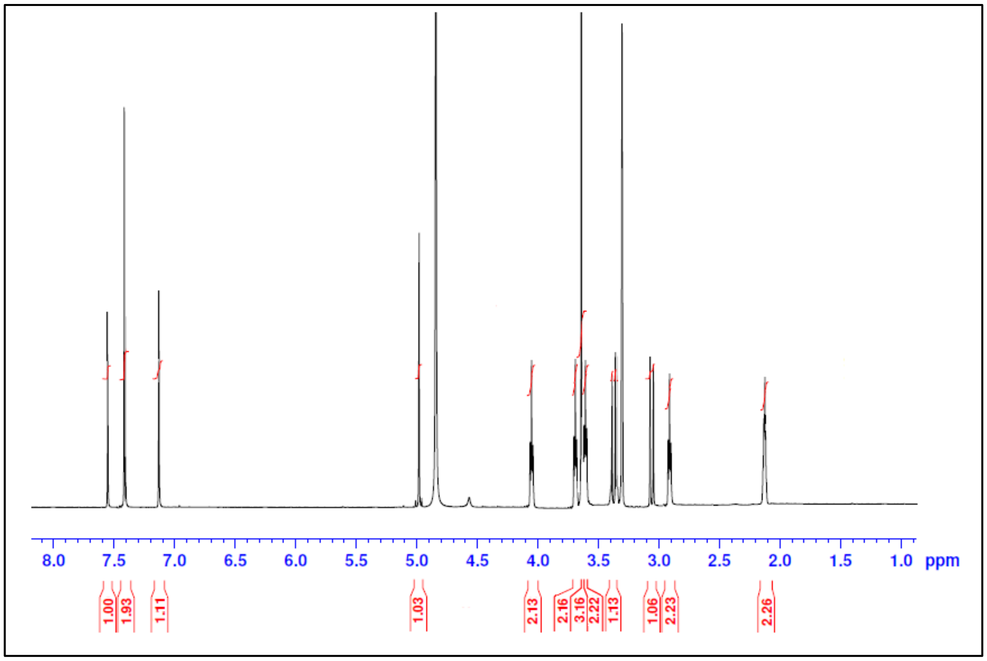

Figure S3. Expansion of ^{1}H NMR spectrum of psammaceratin A (**1**) (MeOD).

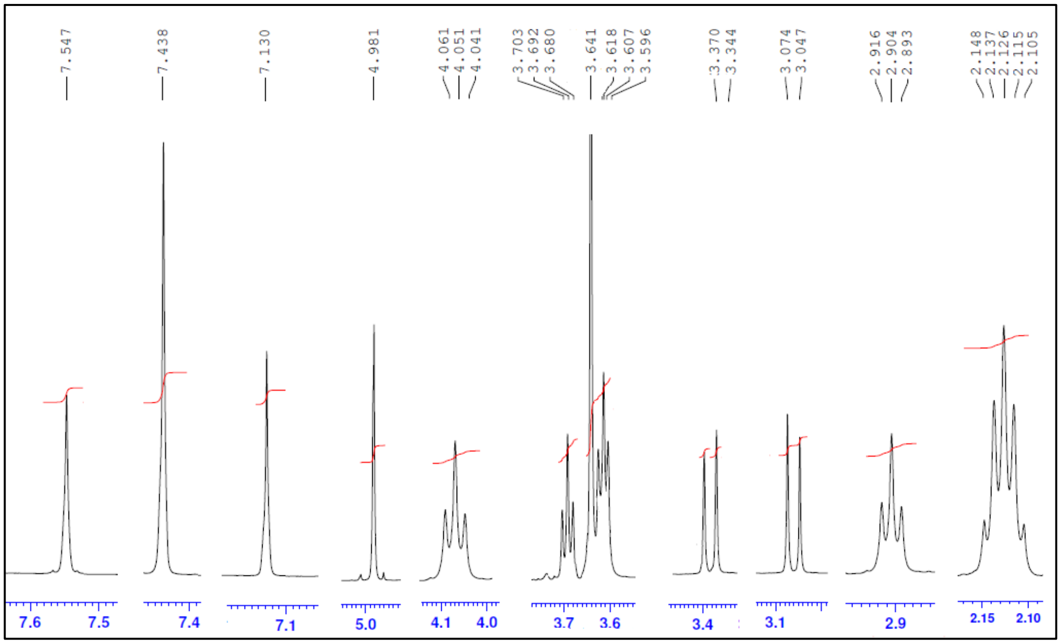

Figure S4. 150 MHz ^{13}C NMR spectrum of psammaceratin A (**1**) (MeOD).

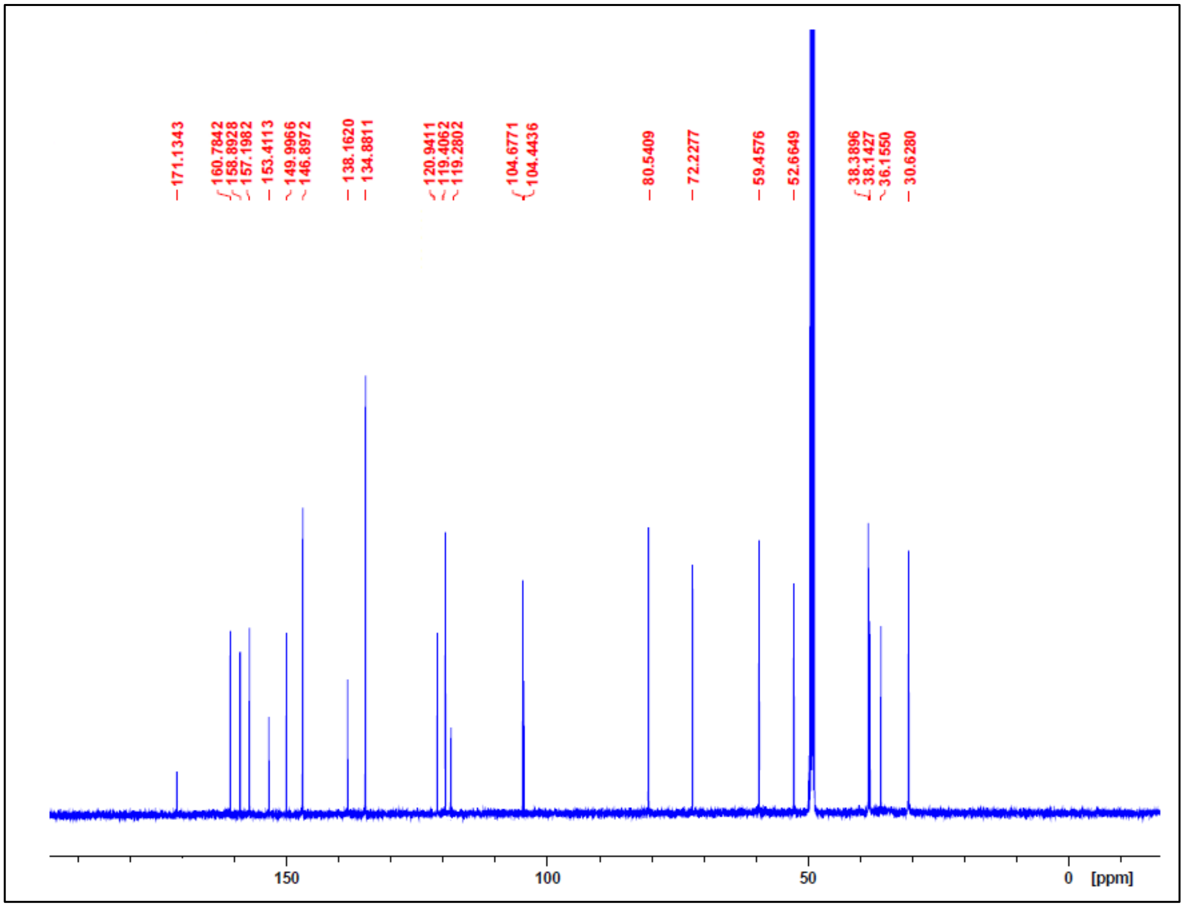

Figure S5. DEPT spectrum of psammaceratin A (**1**) (MeOD).

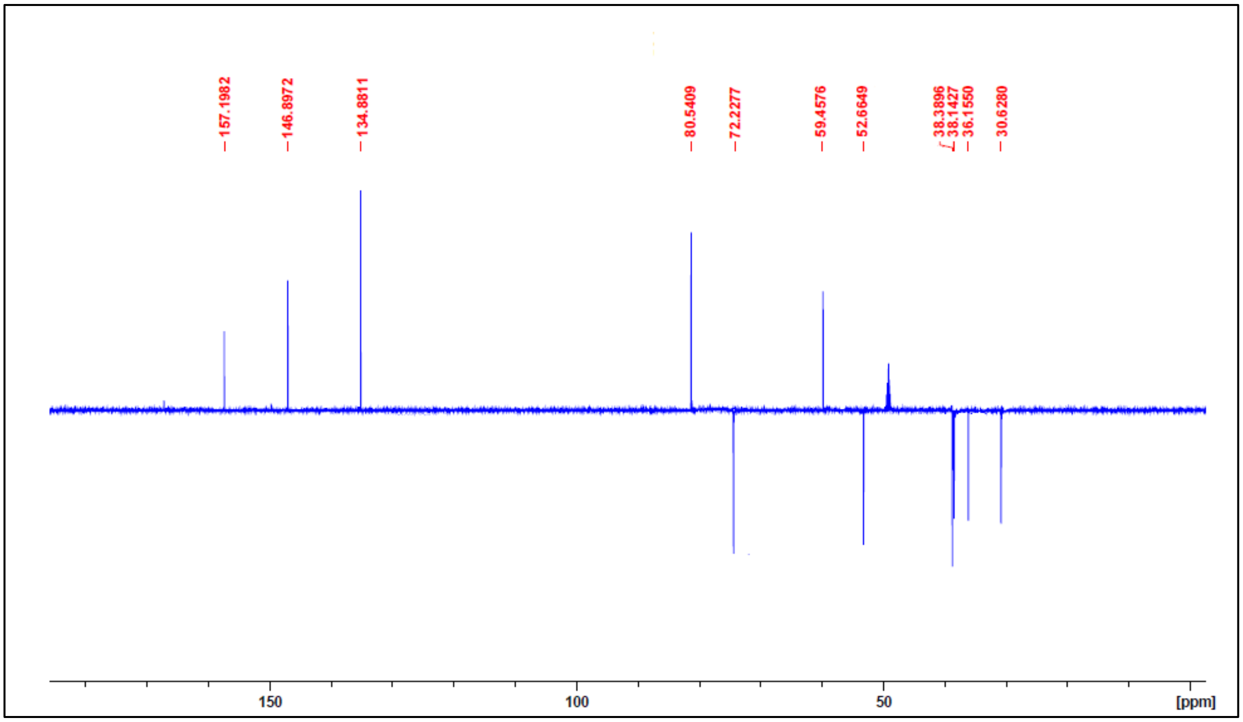

Figure S6. ^{1}H-^{1}H COSY spectrum of psammaceratin A (**1**) (MeOD).

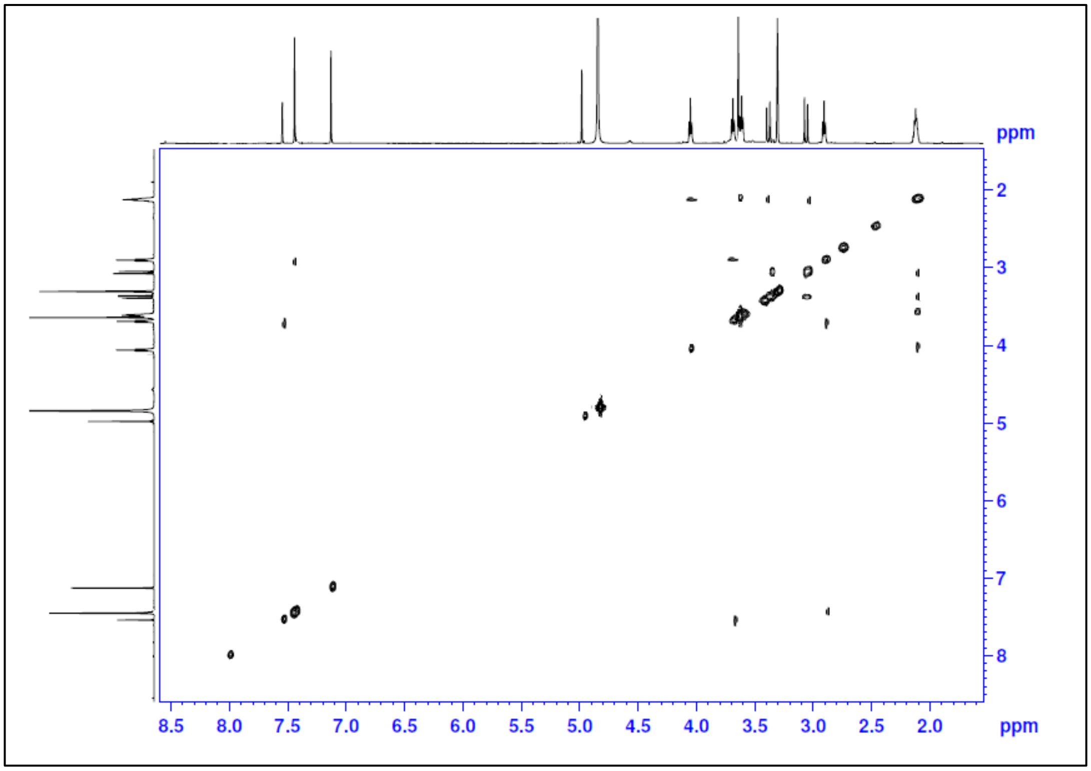

Figure S7. Multiplicity-edited HSQC spectrum of psammaceratin A (**1**) (MeOD).

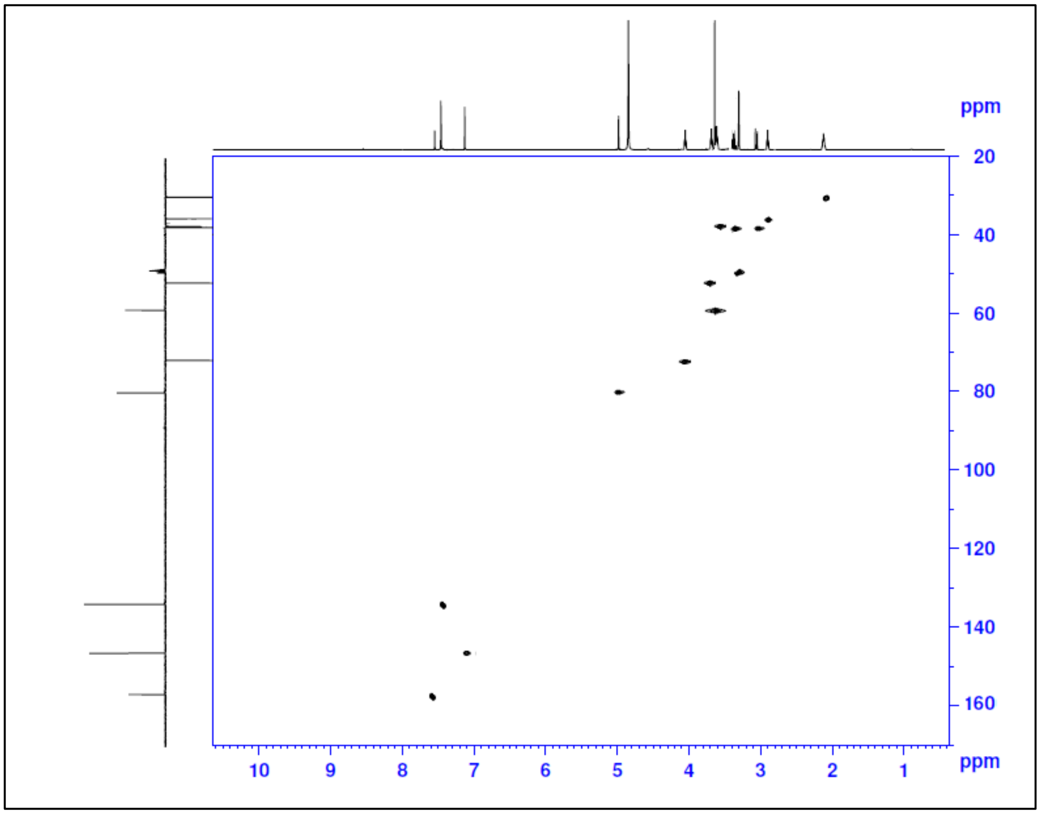

Figure S8. ^{1}H-^{13}C HMBC spectrum of psammaceratin A (**1**) (MeOD).

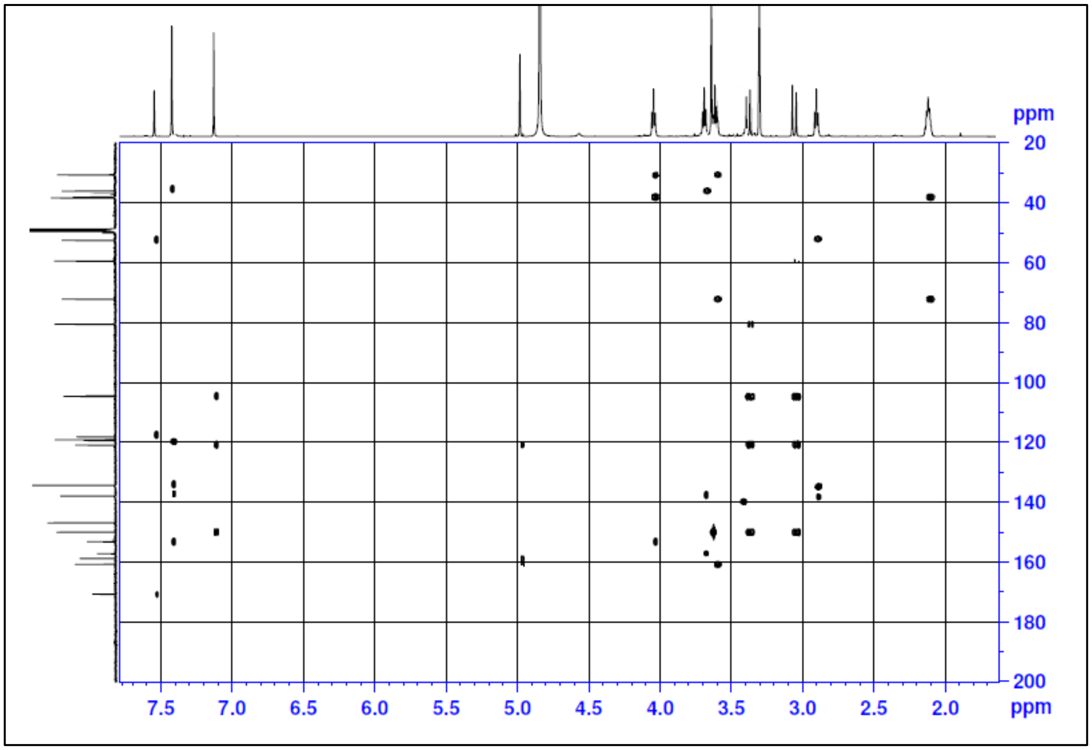

Figure S9. ^{1}H-^{1}H NOESY spectrum of psammaceratin A (**1**) (**1**) (MeOD).

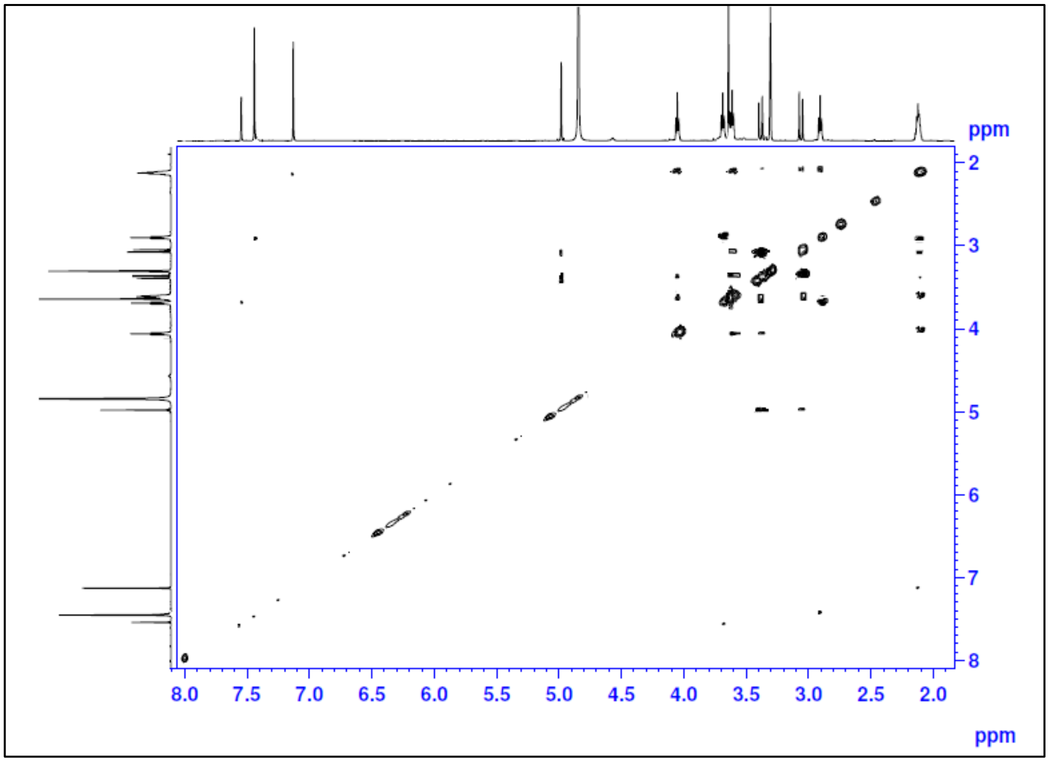

Supplement: Supplementary file 1 [file marinedrugs-19-00433-s001.zip › marinedrugs-1324609-supplementary.pdf]
